# Supplementary material for: Comprehensive Genomic Analysis of Klebsiella pneumoniae and Its Temperate N-15-like Phage: From Isolation to Functional Annotation
Source: Microorganisms. 2025 Apr 15;13(4):908. doi: 10.3390/microorganisms13040908 (PMC12029707; doi:10.3390/microorganisms13040908)
Supplement: Supplementary file 1 [file microorganisms-13-00908-s001.zip › microorganisms-3544195-SI.pdf]

Table S1. **The key findings from the assembly analysis O-antigen and capsular type.**

Category	O1/O2v1 Locus			K64 Locus		
Best Match Locus	O1/O2v1			KL64		
Best Match Type	O2a			K64		
Match Confidence	High-confidence typing confirms serotype assignment accuracy.					
Identity	99.03%			100%		
Coverage	100%			90.10%		
Expected Genes in Locus	7 / 7 (100%)			23 / 24 (95.83%)		
	Gene	Identity %	Coverage %	Gene	Identity %	Coverage %
Expected Gene Details	*wzm*	100%	100%	*galF*	100%	100%
	wzt	100%	100%	*cpsACP*	100%	100%
	wbbM	99.21%	100%	*wzi*	100%	100%
	glf	97.92%	100%	*wza*	100%	100%
	wbbN	97.31%	100%	*wzb*	100%	100%
	wbbO	99.2%	100%	*wzc*	100%	100%
	kfoC	99.54%	100%	*wzx*	100%	81.85%*
				wcoV	100%	23.45%*
				wzy	100%	79.16%*
				wcoU	100%	63.69%*
				wcsF	100%	39.57%*
				wcuK	100%	100%
				wbaZ	100%	100%
				wcaJ	100%	100%
				gnd	100%	100%
				manC	100%	100%
				manB	100%	100%
				rmlB	100	100
				rmlA	100	100
				rmlD	100	100
				rmlC	100	100
				ugd	100	100
Missing Expected Genes	None			KL64_12_*wcoT*		
	Gene	Identity %	Coverage %	Gene	Identity %	Coverage %
Other Serotypes Genes	*manC*	100%	100%	*rfaG*	98.42%	99.74%*
	manB	99.77%	95.65%*	*gmd*	98.80%	100%
	rfbB	98.02%	100%	*HG290*	80.19%	88.25%*
	rfbD	98.65%	100%			
	rfbA	97.19%,	98.62%*			
	rfbC	96.69%	98.37%*			

* Truncated genens

 https://doi.org/10.3390/microorganisms13040908

Table S2. CRISPR-Cas Finder Results for Identified Sequences

This table presents the CRISPR loci identified in two unknown bacterial strains, including their genomic positions, repeat sequences, spacer counts, and conservation levels.

Sequence	Start	End	Length	Consensus Repeat	Repeat Length	Spacer Count	Mean Spacer Size	Repeat Conservation (%)	Spacer Conservation (%)
Seq31	31717	31856	139	TGCCGGGTGGCGGCTGGCGCCTTACCCGGCCTACAAAACCGAAGCAGCAG	50	40	1.00	98	98
Seq37	8742	11391	2649	GTGTTCCCCGCGCCAGCGGGGATAAACCG	29	43	31.95	75.86	95.69

Table S3. Prophages Identified Within the Genome of *Klebsiella pneumoniae* Kpn_R01

Prophages identified within the genome of *K. pneumoniae* Kpn_R01, including their genomic coordinates, length, gene count, and key functional genes. Functional annotations include phage structural proteins, regulators, replication-associated proteins, and DNA packaging proteins.

Prophage ID	Start (bp)	End (bp)	Length (bp)	Genes count	Key Genes and Functions
prophage1	1,402,925	1,440,322	37,397	45	VOG4551 (phage protein), VOG0052 (phage protein), VOG0583 (regulator), VOG058 (regulator)
prophage2	4,089,818	4,130,022	40,204	33	VOG0982 (replication), VOG02 (DNA packaging), VOG4571 (phage protein), VOG4556 (pha protein)
prophage3	4,288,896	4,299,979	1,108	12	VOG0850 (phage protein), VOG0848 (phage protein), VOG0847 (phage protein)
prophage4	4,326,380	4,341,229	14,849	15	VOG6560 (phage protein), VOG4587 (phage protein), VOG1198 (phage protein)
prophage5	4,500,376	4,528,354	27,978	36	VOG3179 (phage protein), VOG1539 (phage protein), VOG5277 (phage protein)
prophage6	5,362,476	5,373,747	11,271	22	VOG3586 (phage protein), VOG6266 (phage protein), VOG7135 (phage protein)
prophage7	5,443,288	5,454,273	10,985	14	VOG3617 (phage protein), VOG2336 (phage protein), VOG2337 (phage protein)
prophage8	5,517,790	5,535,176	17,386	15	VOG0221 (phage protein), VOG9657 (phage protein), VOG1518 (phage protein)
prophage9	5,619,652	5,633,844	14,192	16	VOG8653 (phage protein), VOG2352 (phage protein), VOG6520 (phage protein)
prophage10	5,744,801	5,762,196	17,395	25	VOG5342 (phage protein), VOG4799 (phage protein), VOG3519 (phage protein)
prophage11	5,794,959	5,827,180	32,221	38	VOG1539 (phage tail protein), VOG3179 (phage tail protein), VOG4743 (integrase)

Table S4. Functional annotation of *Klebsiella* phage Kpn_R1 genome.

gene_id	hit.subject	%.idt	aln.len	mis	gap	q.start	q.end	s.start	s.end	evalue	bit.score
Seq1_1	[WP_023339381] MULTISPECIES: plasmid-partitioning protein SopA [Bacteria].	100	387	0	0	1	387	1	387	3.60E-225	790
Seq1_2	[WP_060577942] MULTISPECIES: ParB/RepB/Spo0J family plasmid partition protein [Enterobacteriaceae].	100	325	0	0	1	325	1	325	3.40E-180	640
Seq1_3	[WP_117271415] tail fiber domain-containing protein [Klebsiella pneumoniae].	98.7	382	5	0	1	382	1	382	1.30E-214	755
Seq1_4	[WP_060577940] MULTISPECIES: hypothetical protein [Enterobacteriaceae].	100	215	0	0	1	215	1	215	4.60E-122	446
Seq1_5	[WP_060588407] MULTISPECIES: hypothetical protein [Enterobacteriaceae].	100	102	0	0	1	102	1	102	3.40E-52	213
Seq1_6	[WP_136527542] MULTISPECIES: carbohydrate binding domain-containing protein [Enterobacteriaceae].	96.7	4052	134	0	1	4052	1	4052	0.00E+00	7601
Seq1_7	[WP_057222638] MULTISPECIES: tail assembly protein [Klebsiella].	96.6	203	7	0	1	203	1	203	9.00E-103	381
Seq1_8	[WP_021462612] MULTISPECIES: hypothetical protein [Enterobacteriaceae].	100	116	0	0	1	116	1	116	1.30E-59	238
Seq1_9	[WP_057222639] MULTISPECIES: C40 family peptidase [Klebsiella].	100	236	0	0	1	236	1	236	3.30E-144	520
Seq1_10	[WP_048290175] MULTISPECIES: phage minor tail protein L [Enterobacteriaceae].	100	251	0	0	1	251	1	251	1.40E-146	528
Seq1_11	[WP_017880254] MULTISPECIES: phage tail protein [Enterobacteriaceae].	100	112	0	0	1	112	1	112	9.90E-60	238
Seq1_12	[WP_169534377] phage tail tape measure protein [Klebsiella pneumoniae].	95.3	1118	52	0	1	1118	1	1118	0.00E+00	1995
Seq1_13	[WP_270189497] phage tail protein [Klebsiella variicola].	95	121	6	0	1	121	1	121	2.80E-54	220

Table S5. Genome similarity analysis of *Klebsiella phage Kpn_R1* with other bacteriophages. The table presents comparative genomic data, including sequence identity, alignment length, and taxonomic classification of similar phages.

ID	length	taxid	name	host_group	score	*SG*	%.mean.idt	%.len
Klebsiella phage Kpn_R1	172025	-	user_virus1	-	117181	1	100	100
NC_041899	123490	1912318	Klebsiella phage vB_Kpn_IME260	Pseudomonadota	32377	0.2763	92.8	31.5
NC_042093	111103	2053603	Klebsiella phage Sugarland	Pseudomonadota	30859	0.2633	92.3	30.1
NC_005857	51601	255431	Klebsiella phage phiKO2	Pseudomonadota	22541.5	0.1924	76.8	26.5
NC_048865	109372	2713308	Salmonella phage oldekolle	Pseudomonadota	15462.5	0.132	63.3	21.2
NC_048009	110091	2231354	Salmonella phage S131	Pseudomonadota	15454	0.1319	63.2	21.2
NC_031933	114180	1813782	Salmonella phage 118970_sal2	Pseudomonadota	15381	0.1313	63.9	20.8
NC_071038	110591	2910950	Salmonella phage vB_STy-RN29	Pseudomonadota	15372	0.1312	64.9	20.6
NC_048013	112564	2231351	Salmonella phage S124	Pseudomonadota	15372.5	0.1312	64.9	20.6
NC_048062	114274	2316014	Salmonella phage Sw2	Pseudomonadota	15370.5	0.1312	64	20.8
NC_031902	125114	1813783	Salmonella phage 100268_sal2	Pseudomonadota	15351.5	0.131	64	20.8
NC_048760	114080	2565517	Salmonella phage Sepoy	Pseudomonadota	15279	0.1304	64.1	20.7
NC_048089	114275	2480623	Salmonella phage STG2	Pseudomonadota	15275	0.1304	64.4	20.6
NC_048145	110377	2508069	Salmonella phage 3-29	Pseudomonadota	15261	0.1302	64.8	20.5
NC_024139	108483	1446489	Escherichia phage vB_EcoS_FFH_1	Pseudomonadota	15256.5	0.1302	63.7	20.8
NC_048627	110964	2184265	Escherichia phage SP15	Pseudomonadota	15255	0.1302	63.9	20.7
NC_048000	111601	2041204	Salmonella phage LVR16A	Pseudomonadota	15233	0.13	64.8	20.5
NC_027297	123475	2991861	Salmonella phage Stitch	Pseudomonadota	15234	0.13	64.5	20.5
NC_048005	112582	2231342	Salmonella phage S113	Pseudomonadota	15227.5	0.1299	64.4	20.6
NC_048872	114059	2713277	Salmonella phage atrejo	Pseudomonadota	15224.5	0.1299	64.2	20.7
NC_048012	111447	2231362	Salmonella phage S147	Pseudomonadota	15208.5	0.1298	64.5	20.5
NC_048870	112477	2713279	Salmonella phage bastian	Pseudomonadota	15186.5	0.1296	64.1	20.7
NC_048149	112496	2508061	Salmonella phage 1-23	Pseudomonadota	15191	0.1296	64.3	20.5
